# The effects of acupressure on labor pains during child birth: randomized clinical trial**^1^**

**DOI:** 10.1590/1518-8345.0739.2738

**Published:** 2016-08-08

**Authors:** Reginaldo Roque Mafetoni, Antonieta Keiko Kakuda Shimo

**Affiliations:** 2 Doctoral Student, Faculdade de Enfermagem, Universidade Estadual de Campinas, Campinas, SP, Brazil. RN, Hospital da Mulher "Prof Dr. José Aristodemo Pinotti", CAISM, Universidade Estadual de Campinas, Campinas, SP, Brazil.; 3 PhD, Professor, Faculdade de Enfermagem, Universidade Estadual de Campinas, Campinas, SP, Brazil.

**Keywords:** Acupressure, Complementary Therapies, Labor Pain, Labor, Obstetric

## Abstract

**Objective:**

to analyze the effects of acupressure on the sanyinjiao point for pregnant women in labor at public maternity wards.

**Method:**

single-blind controlled clinical trial, randomly done employing a pragmatic profile. We selected 156 pregnant women in their ≥ 37 week/s, who had cervical dilations of ≥ 4 cm and with two or more contractions in 10 minutes. The pregnant women were randomly divided into three groups at a university hospital in the suburbs of Sao Paulo, Brazil, in order to receive either acupressure treatment, a placebo or participate as part of a control group. The acupressure was applied on the sanyinjiao point during the contractions for 20 minutes. Then the intensity of the pain was evaluated using the Visual Analogue Scale (VAS).

**Results:**

The averages for the pain measured using the VAS were not different for the three groups that were a part of the study (p-value=0.0929), however they were less in the acupressure groups immediately after receiving the treatment (p-value=<0.0001). This was also the case where the treatment lasted for 1 hour (p-value=0.0001). This was the case in comparison with placebo and control groups.

**Conclusion:**

the use of acupressure on the sanyinjiao point is a useful way to alleviate pain in a non-invasive manner. It can improve the quality of care given to pregnant women in labor. Register: RBR-9mhs8r.

## Introduction

Child birth is considered to be a natural phenomenon however the pain that it can bring is both subjective and complex involving physiological, cultural and psychosocial aspects. Currently there are a lot of studies aimed at looking to reduce the pain in child birth (CB) emphasizing non-medicinal treatments that are less invasive and more humanized. These are being developed by nurses and obstetricians. 

In traditional Chinese medicine (TCM) various alternative methods are used to alleviate pain mainly through acupuncture, moxibustion, acupressure and the use of herbs. Acupressure functions in the same way as acupuncture in that it seeks to maintain the balance of energy in the various channels that circulate in the body - called meridians - that are connected to the specific body organs, but without the use of needles. Specific points in the hands and fingers are stimulated (sometimes combining points) to have the best effect in alleviating pain or in putting someone in a relaxed state^4)^. 

The effects of acupressure on the pain during child birth has been studied in controlled and randomized studies (CRS) done in Asian countries^3^ ^,^ ^5^ ^-^ ^6^ and in the Middle East^7^ ^-^ ^12^. Three acupuncture points were identified for acupressure in the CREs: the Sanyinjiao point (SP6), the Hegu point (IG4) and the Zhiyin point (B67). The TCM theory is that these points contain the action that effects the activity in the uterus and which can induce the CB. Advice is also given for it to be used in cases of shoulder dystocia and prolonged CB^6^.

**Figure 1:**
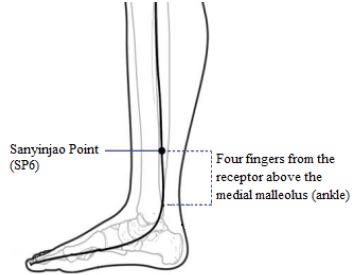
Sanyinjiao Point (SP6)

The SP6 point is believed to have the ability to control some aspects of the reproductive organs being in the case of dystocia and prolonged CB. It is located in the spleen-pancreas meridian, four fingers above the internal malleolus in the front part of the tibia (Figure 1).

Although some studies have associated acupressure on the SP6 point with the reduction in the CB pain, it is not clear if this is the case for uterine dynamics (number and intensity of contractions), which is considered influential in dealing with pain. We did not find Latin American studies that reproduced this technique. The purpose of this study was to analyze the effects of acupressure on the SP6 point for the pain in child birth for expectant mothers in public maternity units.

## Methods

We undertook a controlled and randomized clinical test (based on the consort diagram) employing the use of pragmatism. It was carried out between January and August of 2013. The subjects were pregnant women admitted to a teaching tertiary public hospital on the outskirts of the state of Sao Paulo, Brazil. The study was evaluated and approved by the Ethics Committee on Local Research (Opinion Case Number 182.421). All of the participants in the study agreed to take part in the tests and they signed a consent form demonstrating that they had decided to take part based on their own free will in accordance with the country's laws.

We included expectant mothers of different ages and states from the 37th week in where child birth was expected any time (induced or conduced) having: dilations at ≥ 4 cm, two or more contractions in 10 minutes, skin covering the whole of the SP6 bilateral point and with a live fetus with a cephalic vertex and in good condition. Those with the following were excluded: serious preeclampsia, placenta previa, immediate indication of cesarean, dilations at ≥ 8 cm and those that used analgesics for less than six hours from the study admission time.

The size of the sample was estimated through considering the proposed calculation method for the non-paired^14^ t test. We looked at the percentage differences in the pain using the Visual Analogue Scale (VAS) before and after the treatment in the three studies^6^ ^,^ ^8^ ^,^ ^11^. For the calculations, we adopted a level of significance equal to 5% and a power of 80%, except for studies that also showed results after 60 minutes. In these cases the *Bonferroni* correction was applied at a significant level of 2.5%. The calculations for the sample resulted in the testing of 51 individuals per group which was the biggest size for the test that was calculated amongst the studies, totaling 153 participants.

The pregnant women in labor were allocated through a sequential list of random numbers in blocks of six patients generated using the program Excel(r) and they were distributed in three groups: acupressure (SP6), touch group (TG)/placebo and the control group (CG). The study used the single-blind method (participants in the SP6 and TG groups did not know what group they were in). This was not possible for the CG owning to the actual nature of the study. All of those that were questioned, were identified with a number and their groups were given a letter ensuring that no one could identify each other's group for statistical analysis.

For conducting evaluations on the pain we opted for the VAS model which is a rising scale from 0 10, 0 meaning no pain to 10 which is the worst pain felt. The patients registered their pain using a picture of a grimacing face and writing down a number next to it. The VAS was used and reused immediately after the treatment (VAS20) and 60 minutes after the treatment, once consent had been given by the pregnant women in labor to participate in the study. 

Information in relation to the use of medication and anesthesia was registered in accordance with the relevant procedures and were checked by a team of professionals at the institution. They looked at inpatient records, forms containing the use of anesthesia, partograms and medical prescriptions. The professionals at the units that managed the use of the medications did not know which group each individual belonged to and the researcher had no influence over the prescriptions nor were they manipulated in anyway.

The SP6 group with the pregnant women in labor received deep pressure (± 5kg) with fast decompression applied to their thumbs which did not bring about any discomfort. The TG group received a superficial touch that was of very low intensity (± 100g)^15^. In both groups the contact was at the SP6 bilateral point, during the contractions, in one period of 20 minutes.

The researcher that was responsible for applying the acupressure went through 32 hours' worth of training. A child-cushioned electronic anthropometric scale was used to control and to put consistent pressure on the thumps until the researcher could apply such pressure for the SP6 and TG groups.

The pregnant women in labor obtained normal obstetrical treatment. Where the study was carried out, the following was permitted: the presence of someone to accompany the pregnant lady, the use of methods other than drugs such as taking a shower, massages in the lower back region and breathing exercises as well as liberty of movement (in cases where this did not go against medical advice). 

All of the pregnant women in labor where encouraged during the CB to breath in and out deeply in the direction of the thorax during the contractions and the intervals in orde for their bodies to be totally relaxed (slow thorax-abdomen breathing rhythm)^16^. 

A questionnaire was developed to obtain sociodemographical and clinical data. The content was analyzed in relation to its validity by five experienced analysts who had experience in obstetrics and/or in TCM. No alterations were necessary as it was pre-tested on pregnant women in labor. 

The Kruskal-Wallis test was used to make comparisons between the groups in relation to quantitative variables. The Mann-Whitney test was used in cases where significant differences were found for multiple comparisons. The Chi-squared test was used in relation to the association between groups and variables categories. The Exact Fisher test was used in cases where there were at least 20% of the expected values being less than five. The Friedman test was used for each group in relation to comparisons between the three evaluation periods in the VAS.

 Bonferroni correction was used for the variables where the comparisons between the groups and those that were measured took place in one evaluation period. This divided the significant level by the number of comparisons. The significant level of 1.67% was adopted where the variable was measured in three periods and we compared the groups at every stage. In relation to the VAS variable, aside from comparing the groups at every period we also measured the periods for every group. With this we adopted the significant level of 0.83% for this variable. Lastly for the multiple comparisons, the significant level was corrected, following the same criteria. 

## Results

**Figure 2:**
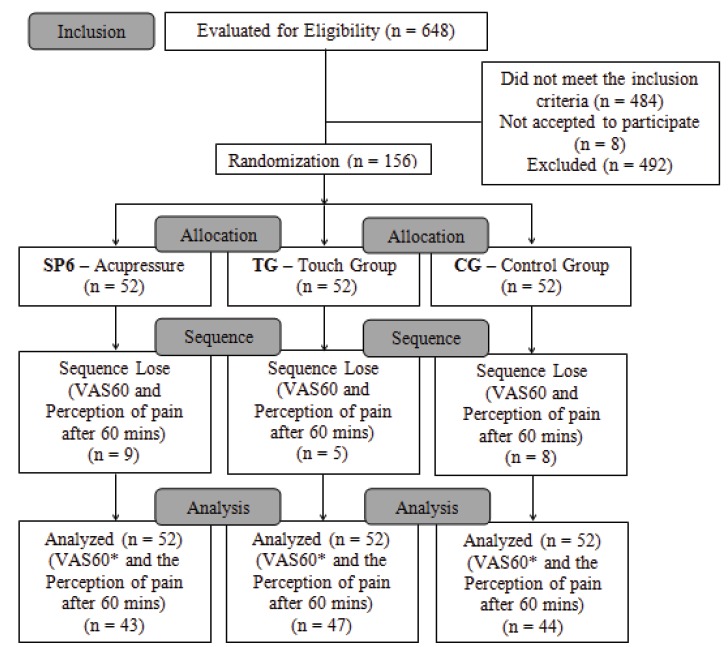
Procedures for the collection of data

156 pregnant women in labor were used in this study and they were distributed equally in three groups. None of them were taken out due to the process of randomization except for the VAS60 variables and based on the perception of pain with 60 minutes owning to the occurrence of analgesia or the birth in the intervals, as per Figure 2.

*VAS60 – the Visual Analogical Scale, after 60 minutes of treatment

In Table 1 are the general characteristics of the pregnant women based on the location of the group. There are no differences between the groups with reference to the variables studied. 

**Table 1:** General characteristics of the women who were studied and their groups. Campinas, SP, Brazil, 2013.

Variable		Groups that were studied			P
		SP6	Touch	Control	
		(N = 52)	(N = 52)	(N = 52)	
Age (years) Average (dp)		26.8(7.1)	26.4(6.4)	25.2(7.3)	0.4567*,†
Level of Schooling (years of study), average (dp)		9.4(2.6)	9.8(2.4)	9.6(3.0)	0.8954*,†
Marital Status, n (%)					
	With a partner	49 (94.2)	50 (96.1)	49 (94.2)	-
	Without a partner	3 (5.8)	2 (3.9)	3 (5.8)	-
Color/ethnicity, n (%)					
	Mixed-race	31 (59.6)	25 (48.1)	24 (46.2)	-
	White	14 (26.9)	22 (42.3)	21 (40.4)	-
	Black	6 (11.6)	5 (9.6)	6 (11.5)	-
	Asian	1 (1.9)	0 (0.0)	1 (1.9)	-
Previous use of Acupressure/Do-in/Shiatsu n (%)					
	Never heard of them	48 (92.3)	44 (84.6)	48 (92.3)	-
	Heard of them, not tried	4 (7.7)	6 (11.5)	4 (7.7)	-
	Heard of them and used them	0 (0.0)	2 (3.9)	0 (0.0)	-
	Number of pre-natal session, average (dp)	9 (3.1)	9.2 (2.5)	9.6 (2.4)	0.4822*,†

* p < 0.05.

†Kruskal-Wallis test

The obstetrical characteristics in Table 2 show the variables of the groups that can influence the responses for pain by the pregnant women.

**Table 2:** General obstetrical characteristics of the pregnant women in the study. Campinas, SP, Brazil, 2013.

Variable			Groups that were studied			P
			SP6	Touch	Control	
			(N = 52)	(N = 52)	(N = 52)	
Number of pregnancies, average (dP)			2.6(1.7)	2.3(1.5)	1.8±1.2	0.0232*,†
	Parity, n (%)					0.2319*,‡
		Nulliparous Women	21(40.4)	28(53.9)	29(55.8)	
		Multiparous Women	31(59.6)	24(46.1)	23(44.2)	
Before the Treatment						
	Amniotic membrane, n (%)					0.0416*,‡
		The whole	26 (50.0)	38 (73.1)	24 (46.2)	
		Artificial Route	11 (21.1)	4 (7.7)	9 (17.3)	
		Spontaneous route	15 (28.9)	10 (19.2)	19 (36.5)	
Cervical dilations (cm), average (dp)			4.9(0.8)	4.65(0.9)	4.6(0.9)	0.1455*,†
Number of contractions, average (dp)			3.3(1.0)	3.19(1.1)	3.4(0.9)	0.6212||,†
Intensity of the contractions, n (%)						0.1693§,¶
	Weak		0 (0.0)	4 (7.7)	2 (3.9)	
	Moderate		25 (48.1)	30 (57.7)	26 (50.0)	
	Strong		27 (51.9)	18 (34.6)	24 (46.1)	
During the Treatment						0.0137||,†
	Nº of contractions in 20 minutes, average (dp)		7,02(1.8)	6.3(2.0)	7.5(2.2)	
After the Treatment						0.4484||,†
	Nº of contractions in 60 minutes, average (dp)		3.6(1.1)	3.4(1.2)	3.6(1.1)	
	Intensity of the contractions, n (%)					0.7995§,¶
		Weak	2 (4.3)	3 (6.1)	4 (8.3)	
		Moderate	20 (42.5)	18 (36.7)	15 (31.3)	
		Strong	25 (53.2)	28 (57.2)	29 (60.4)	
	Amniotic membrane, n (%)					0.0518*,‡
		Artificial Route	10 (19.2)	21 (40.4)	14 (26.9)	
		Spontaneous route	7 (13.5)	6 (11.5)	2 (3.9)	
		Others (cesarean or bust beforehand)	35 (67.3)	25 (48.1)	36 (69.2)	

*p < 0.05 †Kruskal-Wallis test ‡Chi-squared test §p < 0.025 || p < 0.0167 ¶Exact Fisher test.

The De Lee plan, evaluated before the treatment, did not show any differences between the groups. The majority of the pregnant women in labor had a fetus of -3 cm except for two in the TG and two in the CG with -2 cm. The number of contractions referred to during the period of the treatment was different between the groups. The SP6 group and the CG group showed seven contractions compared with six in the TG.

VAS was used to evaluate the pain and for pre-developed questions. The women were able to classify their perception of the pain (Table 3). 

**Table 3:** Difference in the VAS scores and an evaluation of the pain between the groups that were studied. Campinas, SP, Brazil, 2013.

Variable		Groups that were studied				P		
		SP6		Touch		Control		
		(N = 52)		(N = 52)		(N = 52)		
VAS*		Average	dp	Average	dp	Average	Dp	
	Before the Treatment	7.4	1.9	7.1	2.4	7.9	1.9	0.0929^†,‡^
	20 minutes of treatment	5.9	2.3	7.6	2.5	8.5	1.9	<0.0001^†,‡^
	Perception of the pain (20 minutes)	n	%	n	%	n	%	
	Alleviated	34	65.4	7	13.5	1	1.9	
	No change	17	32.7	22	42.3	24	46.2	<0.0001^§,||^
	Worse	1	1.9	23	44.2	27	51.9	
		(N = 43)		(N = 47)		(N = 44)		
VAS*		Average	dp	Average	dp	Average	Dp	
	60 minutes of treatment	6.5	2.2	8.1	2.3	8.8	1.8	<0.0001^†,‡^
	Perception of the pain (60 minutes)	n	%	n	%	n	%	
	Alleviated	9	20.9	4	8.5	0	0.0	
	No change	26	60.5	12	25.5	14	31.8	0.0018^†,¶^
	Worse	8	18.6	31	66	30	68.2	

*Visual Analogical Scale

† p < 0.0083 §p < 0.0167.

Kruskal-Wallis test ‡Chi-squared test § ¶Exact Fisher test.

There were statistical differences (p<0.0001, Fridman test) in the VAS values for each group for the three times when the evaluations were done. This came about due to the rise in the TG and CG averages in relation to their being presented before the treatment. This was not the case of the SP6 group whose values were reduced after the acupressure. When the groups are compared (in in accordance with the Mann-Whitney test) the difference can be seen immediately, with 60 minutes of treatment between the SP6 group in relation to the TG and CG groups (p<0.0001). There were no significant differences in the two moments that were analyzed (p=0.0551 for 20 minutes and p=0.1287 for 60 minutes). 

In relation to intravenous analgesics or those that were intra-musculary administered during the CB, the number was small due to their use being discouraged in obstetric wards. The medications used were either: dipyrone 1gr, meperidine 50mg or tramadol 50 mg. They were used on two women in the TG, two in the CG and only one in the SP6 group. 

The analgesia epidural or the spinal anesthesia were available for women depending on their need. Their use was as follows: 69.23% in the SP6 group (n 36) and 79.92% (n 40) in the other groups (Chi-squared test, p=0.5840). Cervical dilations were registered when the anesthesia was applied. The anesthesia was chosen by the team of anesthetists. There were no differences in the groups (p=0.6650, Kruskal-Wallis test) showing a median of 6 cm for those in the SP6 group and 5 cm for those in the CG.

There were no differences between the averages in the first (p=0.9542) and fifth minute of life for the newly born babies when we used the Apgar score (SP6 9.62 verses TG 9.54 verses TG 9.29, p=0.7218). Therefore this indicator was not changed for this type of treatment for all the groups.

## Discussion

At the admission of the pregnant women in this study before the start of the treatment, there was a difference in the number pregnancies. However, this did not affect the other variables which can be considered influences on their responses to the pain. These variables included: parity, the integrity of the amniotic membrane, the cervical dilations and the number and intensity of the contractions. We noted during the treatment fewer contractions in the TG group in relation to the SP6 and CG group that received the acupressure.

Through an evaluation of the pain we saw a significant reduction in the group that received acupressure in the SP6 point in comparison with the TG and CG groups. The average for the VAS was less in the two evaluations after the treatment in the SP6 group. This data is similar to the previous CRS^7^ ^,^ ^10^ ^)^ that evaluated the point. There was a study where acupressure was applied for 30 minutes during contractions and for 20 minutes. It was applied on the SP6 point and for another group on the IG4 point. There were no differences for the VAS in the SP6 and IG4 groups, but there were differences when compared to the CG which was 8 cm in dilations. When the dilations were complete, we did not see any differences. This was noted at the CRS in South Korea where the dilations were between 9 and 10 cm. The points were close for the CG.

The other three CRS^3^ ^,^ ^6^ ^,^ ^8^ evaluated the SP6 point on pain. The VAS score was not subsequently inferior to the base values. Its rise, however, was greater for the women with the placebo. These studies did not include a third group. Pain in the CB is gradual. Also the fact that the pain did not get worse shows the effectiveness of the care given.

The participants in the TG showed, on average, a lower rise in the VAS even though on the whole there were no significant differences. This may be due to the superficial touch on the SP6 bilateral point which is a common practice for palpation points and body energy points that stimulates the flux of energy^3^. Continuous support for pregnant women in labor can reduce complaints of birth pains, favoring the development of the CB as note in a revision of Cochrane^18^. 

The effect of acupressure on pain is not entirely clear physically and biochemically speaking. An explanation based on TCM called the "G*ate-control theory of pain"* was given in 1965 which talks about cutaneous tactile stimulation used to alleviate pain^19^. The theory states that acupressure activates mechanoreceptors that provoke the thick fibers (A-alfa and A-beta) and which direct them to shut the door whilst the fine fibers (A-delta and C) direct the others to open them. This open and closing of doors represents the transmission or not of the the pain along the nerves depending on the intensity of the pressure. This then stops the transmission of the pain to the spine^7^. Some authors suggest deep massages in the seven acupuncture points during contractions and light massages in the lumbar region in the intervals in order to activate the thick fibers (closing the door). This will inhibit the pain and reduce discomfort for the lady that receives the treatment^20^.

The women did not use a lot of intravenous or intra-muscular analgesic in this study. We also noted this in other previous studies^6^. They were used a great deal for all the women in the three CRSs^7^ ^,^ ^11^ ^,^ ^21^ whilst in other studies the analgesics were not mentioned or it was not clear if they had been used. In the three groups for the CRS, the analgesia epidural and its related combination analgesia was used a lot. There were less of these resources for the SP6, TG and CG groups, however the statistical analysis did not show any differences. If we consider and accept the reduction in score for the pain in the first 20 minutes, the replication of the treatment during the CB could slow or reduce the use of analgesia or other forms of early interventions allowing for a natural child birth.

There were no alterations in the Apgar scores between neonate women that received acupressure in the SP6 point compared to the other participants. Thus there were no adverse effects on the neonate women that received this intervention which corroborated with the CRS^7^ ^,^ ^10^ where the variable was evaluated.

A limitation of this study was the use of the single-blind method due to the lack of an external evaluator after the treatment. Note that this in itself did not influence or have any bearing on the responses to the variables studied. Another limitation for was the use of manual palpation to assess the intensity of the contractions. The idea was not to use an invasive method that could result in discomfort for the women. The third limitation was the use of a university hospital which was hospital renowned for treating high risk pregnancies. This may have contributed to the high rates of medical interventions.

## Conclusion

The results show that the use of acupressure on the SP6 point is a complementary method that is non-invasive and can alleviate pain during childbirth, without presenting any adverse effects for pregnant women in labor and neonate women.

Acupressure is a useful way to alleviate pain and it can be easily be put into practice in medical institutions with a view to improving the quality of care given to pregnant women in labor who would like a natural birth. However the effect of the treatment in the reduction of pain is small, which suggests that acupressure may be more effective where there are cervical dilations up to 8 cm and there is cephalic presentation. 

Acupressure is an alternative for women that prefer to use methods that do not involve drugs and side effects which is often the case for medical practitioners in obstetrics.
